# Mirror-image polymerase chain reaction

**DOI:** 10.1038/celldisc.2017.37

**Published:** 2017-10-17

**Authors:** Wenjun Jiang, Baochang Zhang, Chuyao Fan, Min Wang, Jiaxing Wang, Qiang Deng, Xianyu Liu, Ji Chen, Jishen Zheng, Lei Liu, Ting F Zhu

**Affiliations:** 1 School of Life Sciences, Tsinghua-Peking Joint Center for Life Sciences, Center for Synthetic and Systems Biology, Ministry of Education Key Laboratory of Bioinformatics, Tsinghua University, Beijing, China; 2 Tsinghua-Peking Joint Center for Life Sciences, Ministry of Education Key Laboratory of Bioorganic Phosphorus Chemistry and Chemical Biology, Department of Chemistry, Tsinghua University, Beijing, China; 3 School of Life Sciences, University of Science and Technology of China, Hefei, China

**Keywords:** chirality, D-amino acid, L-DNA, mirror-image polymerase chain reaction (miPCR), *Sulfolobus solfataricus* P2 DNA polymerase IV (Dpo4)

## Abstract

The construction of mirror-image biological systems may open the next frontier for biomedical technology development and discovery. Here we have designed and chemically synthesized a mutant version of the thermostable *Sulfolobus solfataricus* P2 DNA polymerase IV (Dpo4) consisting of d-amino acids. With a total peptide length of 358 amino acid residues, it is the largest chemically synthesized d-amino acid protein reported to date. We show that the d-polymerase is able to amplify a 120-bp l-DNA sequence coding for the *Escherichia coli* 5S ribosomal RNA gene *rrfB* by mirror-image polymerase chain reaction, and that both the natural and mirror-image systems operate with strict chiral specificity. The development of efficient miPCR systems may lead to many practical applications, such as mirror-image systematic evolution of ligands by exponential enrichment for the selection of therapeutically promising nuclease-resistant l-nucleic acid aptamers.

## Introduction

We have previously reported the chemical synthesis of a 174-residue d-amino acid African Swine Fever Virus polymerase X (ASFV pol X) system [1], in which we demonstrated that two processes in the central dogma of molecular biology, the template-directed replication of DNA and transcription into RNA, could be catalyzed by a d-amino acid polymerase on an l-DNA template. The establishment of this mirror-image genetic replication and transcription system was a small step towards chemically synthesizing an alternative, mirror-image form of life in the laboratory [2, 3], whereas achieving this ultimate goal requires many more efficient molecular tools such as a thermostable enzyme capable of mirror-image polymerase chain reaction (miPCR).

Advances in solid-phase peptide synthesis (SPPS) and native chemical ligation have enabled the synthesis of a number of d-amino acid proteins by ligating short, synthetic d-peptide segments into longer ones [1, 4, 5]. While the chemical synthesis of most PCR enzymes such as the 832-residue *Taq* polymerase might be still beyond current technology, the synthetic route for synthesizing a small thermostable polymerase, such as the 352-residue *Sulfolobus solfataricus* P2 DNA polymerase IV (Dpo4), has been independently developed by us and others [6, 7]. Here we have chemically synthesized a mutant version of the thermostable Dpo4 with d-amino acids by further improving our previously reported synthetic route [6]. We show that the mutant d-polymerase (d-Dpo4-5m) is able to amplify a 120-bp l-DNA sequence coding for the *Escherichia coli* 5S ribosomal RNA (rRNA) gene *rrfB* by miPCR.

## Results

### Chemical synthesis and folding of d-Dpo4-5m

**Figure 1:**
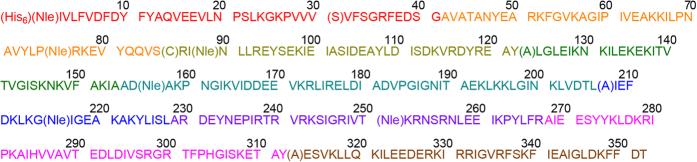
d-Dpo4-5m amino acid sequence. d-Dpo4-5m amino acid sequence with five point mutations (C31S, S86C, N123A, S207A and S313A; highlighted by parentheses) and an N-terminal d-His_6_ tag. Isosteric Nle (highlighted by parentheses) was used to replace all the methionine residues (Met1, Met76, Met89, Met157, Met216 and Met251).

**Figure 2:**
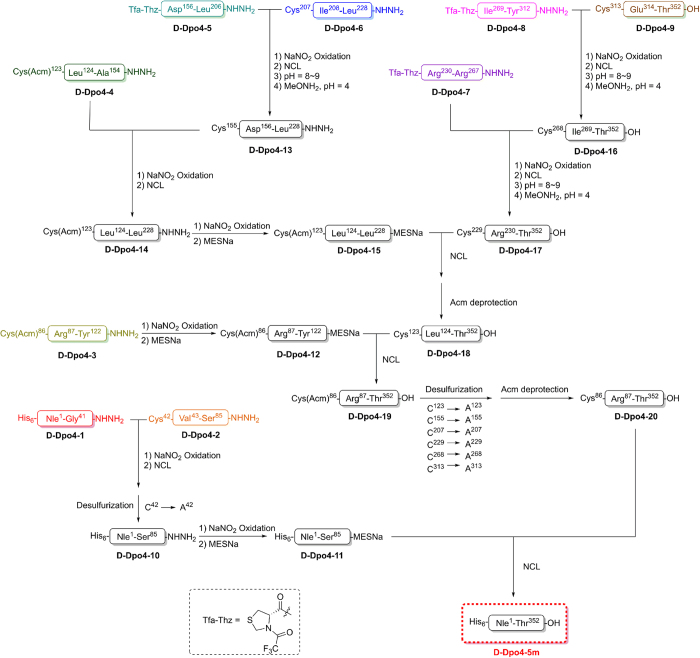
Synthetic route for d-Dpo4-5m. Total chemical synthesis of d-Dpo4-5m by assembling 9 peptide segments in the C- to N-terminus direction using hydrazides as thioester surrogates.

We carried out the chemical synthesis of a d-amino acid version of the mutant Dpo4 protein with improvements to our previously reported synthesis strategy [6] by replacing the S86A mutation with S86C (Figure 1), and by performing stepwise desulfurization (Figure 2). We show that the PCR efficiency of the mutant Dpo4 (Dpo4-5m) is comparable to that of the recombinant wild-type (WT) Dpo4 purified from *E. coli*, suggesting that these five point mutations (C31S, S86C, N123A, S207A and S313A) do not significantly affect the PCR efficiency of the enzyme (Supplementary Figure S1). Furthermore, we replaced all the methionine residues (Met1, Met76, Met89, Met157, Met216 and Met251) with isosteric norleucine (Nle) to avoid potential oxidation to the methionine residues in the SPPS and peptide ligation processes [6, 8], and added a d-polyhistidine tag (d-His_6_, which we show can also bind to a nickel column) at the N-terminus of the synthetic enzyme to facilitate the downstream protein purification, making the total length of the synthetic peptide to 358 amino acid residues (Figure 1).

We chemically synthesized the 9 peptide segments with d-amino acids, and assembled the segments in the C- to N-terminus direction by native chemical ligation using hydrazides as thioester surrogates [9–13] (Figure 2; Supplementary Figures S2–S16). The full-length d-protein was analyzed by reversed-phase high-performance liquid chromatography (RP-HPLC) (Supplementary Figure S16B) and electrospray ionization mass spectrometry (ESI-MS) (Supplementary Figure S16C; observed M.W. 40 832.0 Da, calculated M.W. 40 832.6 Da). Then the d-protein was folded by successive dialysis against a series of renaturation buffers containing 4 m, 2 m, 1 m, 0.5 m, 0.25 m, and 0 m Gn·HCl, respectively.

**Figure 3:**
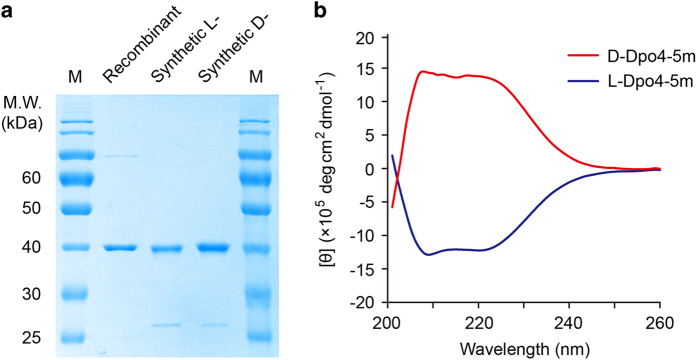
Biochemical characterization of Dpo4-5m. (**a**) Recombinant Dpo4-5m purified from the *E. coli* strain BL21(DE3), as well as chemically synthesized 40.8 kDa l- and d-Dpo4-5m were analyzed by 12% SDS-PAGE, stained by Coomassie Brilliant Blue. A small fraction of unligated peptide segments can be observed in the synthetic l- and d-Dpo4-5m. M, protein marker. (**b**) CD spectra of the synthetic l- and d-Dpo4-5m. The CD curves were averaged from three independent measurements and background-subtracted.

Next, we heated the folded D-polymerase to 78 °C to precipitate the thermolabile peptides, which were removed by ultracentrifugation, followed by nickel column purification (Materials and Methods). The l-polymerase (synthesized with l-amino acids, purified and folded by the same procedures) and d-polymerase were analyzed by sodium dodecyl sulfate-polyacrylamide gel electrophoresis (SDS-PAGE) (Figure 3a), and by circular dichroism (CD), showing that the chemically synthesized and folded l- and d-proteins were mirror images of each other (Figure 3b).

### miPCR by synthetic d-Dpo4-5m

**Figure 4:**
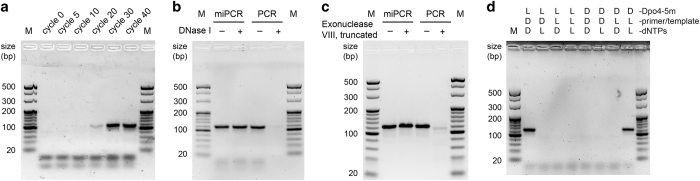
miPCR by d-Dpo4-5m. (**a**) PCR amplification of a 120-bp l-DNA sequence by synthetic d-Dpo4-5m, performed in 50 mm HEPES (pH 7.5), 5 mm MgCl_2_, 50 mm NaCl, 0.1 mm EDTA, 5 mm DTT, 10% glycerol, 3% DMSO, 0.1 mg ml^−1^ BSA, 100 μm (each) l-dNTPs, 0.5 μm (each) l-primers, 20 nm l-template, and ~500 nm d-Dpo4-5m polymerase for 40 cycles. The products were analyzed by 3% sieving agarose gel electrophoresis and stained by GoldView, with cycle numbers from which they were sampled indicated above the lanes. (**b**,**c**) The products of miPCR and PCR were digested by DNase I and Exonuclease VIII, truncated, respectively, analyzed by 3% sieving agarose gel electrophoresis and stained by GoldView. (**d**) Chiral specificity assay with different (a total of 8) chiral combinations of l- or d-polymerases, d- or l-DNA primer/template pairs, and d- or l-dNTPs, analyzed by 3% sieving agarose gel electrophoresis and stained by GoldView. M, DNA marker.

Having synthesized and folded the synthetic d-Dpo4-5m, we carried out miPCR with a synthetic 120-nt l-DNA template coding for the *E. coli* 5S rRNA gene *rrfB*, L-DNA primers (Supplementary Table S1), and l-deoxyribonucleotide triphosphates (l-dNTPs) in an achiral buffer (50 mm HEPES at pH 7.5). We show that the synthetic d-polymerase is able to amplify the l-DNA sequence after up to 40 cycles using a regular PCR program (including denaturation, annealing, and extension steps; see Materials and Methods). The miPCR products resulted in a clear band in the agarose gel with the expected length of 120 bp, which increased in intensity with cycle numbers (Figure 4a). However, the miPCR system appeared to be less efficient than the natural one (requiring a longer extension time), which is likely due to the impurities in the l-dNTPs as observed in previous studies on the ASFV pol X system [1].

The products of miPCR and PCR were digested by an endonuclease (DNase I) and exonuclease (Exonuclease VIII, truncated), in which case the products of miPCR were entirely resistant to digestion by both endonuclease and exonuclease of natural chirality (Figure 4b and c). This feature of amplified L-DNA sequences makes them promising candidates for *in vivo* applications, eg, as nuclease-resistant l-nucleic acid aptamers for research and therapeutic purposes [14, 15]. We then estimated the fidelity of the d-Dpo4-5m system using a corresponding l-polymerase on a D-DNA template [6], as a practical l-DNA sequencing technology is currently unavailable. The error rate of the Dpo4-5m system measured in the order of 10^−4^, consistent with the replication error rate of the WT Dpo4 reported in previous studies [16, 17].

### Chiral specificity of l- and d-Dpo4-5m PCR systems

Next, we investigated whether the natural and the mirror-image Dpo4-5m could operate with strict chiral specificity. We tested their chiral specificity with different (a total of 8) chiral combinations of l- or d-polymerases, d- or l-DNA primer/template pairs, and d- or l-dNTPs, and analyzed the PCR amplification products by agarose gel electrophoresis (Figure 4d). We show that the PCR amplification only occurred with l-Dpo4-5m on a d-DNA primer/template pair supplied with d-dNTPs (the natural system), and with d-Dpo4-5m on an l-DNA primer/template pair supplied with l-dNTPs (the mirror-image system), but not with any of the other combinations. These results suggest that both the natural and mirror-image Dpo4-5m systems have strict chiral specificity, which was also observed in previous studies on the ASFV pol X system [1].

## Discussion

Here we have chemically synthesized a mutant version of the thermostable Dpo4 with d-amino acids, and shown that the d-polymerase is capable of amplifying a 120-bp l-DNA sequence coding for the *E. coli* 5S rRNA gene *rrfB* by miPCR. The development of efficient miPCR systems may lead to various future applications. For example, it may enable mirror-image systematic evolution of ligands by exponential enrichment (miSELEX) for the direct selection of l-nucleic acid aptamers against biological targets as potential research and therapeutic tools [14, 15]. However, DNA polymerases often show lower PCR performance with GC-rich templates, and this may also be true with Dpo4 based on previous kinetic studies on the enzyme [18], whereas many DNA aptamers display G-quadruplex structures and are GC-rich [19]. In addition, in our experience, the system was also less efficient with lower amounts (<5 nm) of input l-DNA templates. Thus improving the miPCR efficiency and reducing its preference to template sequences in the randomized DNA pool is crucial for realizing miSELEX.

The next big challenge in the effort to build a complete mirror-image central dogma is the construction of a mirror-image ribosome [20, 21]. Because both the ASFV pol X and Dpo4 systems suffer from low amplification efficiency (especially with DNA templates longer than several hundred bp) and poor fidelity [1, 6, 16, 17], to obtain the mirror-image rRNAs of up to a couple of kilobases requires a genetic replication and transcription system with efficiency and fidelity much higher than the current ones. An important technical issue to resolve here is the quality of l-dNTPs, which likely have affected the efficiency of the enzymatically catalyzed mirror-image polymerization [1]. Other potential strategies towards realizing more efficient miPCR include to look beyond Dpo4 for other thermostable polymerases in nature, and to apply directed evolution approaches to search for more efficient polymerase mutants [22–24]. Another hurdle in the construction of mirror-image rRNAs is the lack of long l-DNA template sequences in nature, although the ability to perform assembly PCR with short chemically synthesized oligonucleotides may provide a partial solution [6, 7].

While we were preparing this work, an alternative strategy for synthesizing the d-amino acid Dpo4 with 3 cysteine mutations has been reported [7], in which an average peptide segment length of ~70 amino acid residues was required for the total chemical synthesis. While the strategy reported here required more point mutations to be made (a total of 5 point mutations to the WT Dpo4), an average peptide segment length of ~40 residues was applied (which is more accessible for most laboratories with current SPPS technology [25]). In addition, we added a d-polyhistidine tag (d-His_6_) at the N-terminus of the synthetic d-polymerase. While this approach has slightly increased the total size of the synthetic d-protein for chemical synthesis, it has made the downstream purification of the d-polymerase more convenient to carry out. Furthermore, we replaced all the Met residues with isosteric Nle to avoid potential oxidation to the Met residues in the SPPS and protein ligation processes [6]. We also optimized the purification methodology for the synthetic d-polymerase by heating the folded d-protein to remove the thermolabile peptides, which greatly helped to eliminate the unligated peptide segments and misfolded proteins. Synthesizing an l-amino acid version of the protein instead of only the d-protein also helped us to adjust and optimize the synthetic route with much lower cost [6], and allowed us to compare the CD spectra of the chiral twins.

## Materials and methods

### Materials

Fmoc-D-Thr(t-Bu)-Wang resin was purchased from GL Biochem Co., Ltd (Shanghai, China). 2-Chlorotrityl chloride resin was purchased from Tianjin Nankai Hecheng Science & Technology Co., Ltd (Tianjin, China). 9-Fluorenylmethyl carbazate (Fmoc-NHNH_2_) was purchased from Adamas Reagent Co., Ltd (Shanghai, China). Fmoc-d-amino acids were purchased from GL Biochem Co., Ltd and BO MAI JIE Technology Co., Ltd (Beijing, China). Trifluoroacetic acid (TFA), *N*,*N*-dimethylformamide (DMF), thioanisole, triisopropylsilane (TIPS), sodium 2-mercaptoethanesulfonate (MESNa), *O*-methylhydroxyamine hydrochloride, PdCl_2_, silver acetate (AgOAc), and 2,2′-azobis[2-(2-imidazolin-2-yl)propane] dihydrochloride (VA-044) were purchased from J&K Scientific Ltd (Beijing, China). 4-Mercaptophenylacetic acid (MPAA) was purchased from Alfa Aesar Chemicals Co., Ltd (Shanghai, China). Tris(2-carboxyethyl)phosphine hydrochloride (TCEP·HCl) was purchased from Tianjin Liankuan Fine Chemical Co., Ltd (Tianjin, China). 1,2-Ethanedithiol (EDT) was purchased from TCI Development Co., Ltd (Shanghai, China). Piperidine, Na_2_HPO_4_·12H_2_O, and Et_2_O were purchased from Sinopharm Chemical Reagent Co., Ltd (Shanghai, China). Guanidine hydrochloride (Gn·HCl), NaOH, NaH_2_PO_4_·2H_2_O, hydrochloric acid, acetic acid, and NaCl were purchased from Sinopharm Chemical Reagent (Beijing, China). Dichloromethane (DCM) and NaNO_2_ were purchased from Beijing Chemical Works (Beijing, China). Boc-Cys(Acm)-OH, 1-hydroxybenzotriazole (HOBt) anhydrous, and *O*-(6-chlorobenzotriazol-1-yl)-*N*,*N*,*N′*,*N′*-tetramethyluronium hexafluorophosphate (HCTU) were purchased from GL Biochem Co., Ltd (Shanghai, China). *N*,*N*-Diisopropylethylamine (DIEA) was purchased from Beijing Ouhe Technology Co., Ltd (Beijing, China). Ethyl cyanoglyoxylate-2-oxime (Oxyma), *N*,*N′*-diisopropylcarbodiimide (DIC), and DL-1,4-dithiothreitol (DTT) were purchased from Adamas Reagent Co., Ltd (Shanghai, China). Acetonitrile (HPLC grade) was purchase from J. T. Baker (Phillipsburg, NJ, USA). l-deoxynucleoside phosphoramidites and l-dNTPs were purchased from ChemGenes (Wilmington, MA, USA).

### Fmoc-based solid-phase peptide synthesis

All peptides were synthesized by Fmoc-based SPPS manually or using the CSBio CS336S peptide synthesizer (Menlo Park,CA, USA). d-Dpo4-9 with C-terminal carboxylate was elongated on Fmoc-D-Thr(t-Bu)-Wang resin. The other eight segments (d-Dpo4-1 to d-Dpo4-8) were synthesized on Fmoc-hydrazine 2-chlorotrityl chloride resin to obtain peptide hydrazides [26]. A pseudoproline dipeptide [27] was incorporated at position Phe337-Ser338 (in segment d-Dpo4-9). Three isoacyl dipeptides [28] were incorporated at positions Val30-Ser31 (in segment d-Dpo4-1), Ala102-Ser103 (in segment d-Dpo4-3) and Ile144-Ser145 (in segment d-Dpo4-4), respectively. All resins were swelled in DCM/DMF for 30 min before deprotection. The Fmoc groups of both the resin and the assembled amino acids were removed by treating with 20% piperidine and 0.1 mol l^−1^ HOBt in DMF twice (5 and 10 min, respectively). The resin was washed with DMF and DCM thoroughly before all deprotection and subsequent condensation reactions. In the manual condensation reactions, Arg, His and Cys were coupled for 1 h with 4 equiv. Fmoc-amino acids, 3.8 equiv. HCTU, and 8 equiv. DIEA at 30 °C to avoid side reactions at high temperature. Other amino acids, the pseudoproline dipeptide and isoacyl dipeptides were coupled for 20 min at 75 °C using 4 equiv. Fmoc-amino acids or 3 equiv. Fmoc-dipeptides, 4 equiv. Oxyma, and 4 equiv. DIC. Peptide synthesis by automatic synthesizer was carried out for 30 min at 60 °C with 3–4 equiv. Fmoc-amino acids, 4 equiv. Oxyma, and 4 equiv. DIC. After all the amino acids were assembled, the remaining Fmoc group was removed and a cleavage cocktail (TFA/H_2_O/TIPS/thioanisole/EDT, 82.5/5/5/5/2.5) was added. The cleavage reaction took 3 h under agitation at 30 °C. Most of the TFA in the mixture was removed by N_2_ blowing, and cold ether was added to precipitate the crude peptide. After centrifugation, the supernatant was discarded and the precipitates were washed twice with ether. The crude peptides were dissolved in CH_3_CN/H_2_O, analyzed by RP-HPLC and ESI-MS, and purified by semi-preparative RP-HPLC.

### Native chemical ligation

The C-terminal peptide hydrazide segment was dissolved in acidified ligation buffer (aqueous solution of 6 m Gn·HCl and 0.1 m NaH_2_PO_4_, pH 3.0). The mixture was cooled in an ice-salt bath (−15 °C), and 10–20 equiv. NaNO_2_ in acidified ligation buffer (pH 3.0) was added. The activation reaction system was kept in ice-salt bath under stirring for 30 min, after which 40 equiv. MPAA in ligation buffer and 1 equiv. N-terminal Cys peptide were added, and the pH of the solution was adjusted to 6.5 at room temperature. After overnight reaction, 100 mm tris(2-carboxyethyl)phosphine hydrochloride (TCEP·HCl) in pH 7.0 ligation buffer was added to dilute the system twice and the reaction system was kept at room temperature for 1 h under stirring. Finally, the ligation product was analyzed by RP-HPLC and ESI-MS, and purified by semi-preparative RP-HPLC.

### Reversed-phase high-performance liquid chromatography, electrospray ionization mass spectrometry and CD

All RP-HPLC analyses and purifications were carried out using Shimadzu Prominence HPLC systems with SPD-20A UV-Vis detectors and LC-20AT solvent delivery units. Ultimate XB-C4 column (Welch, 5 μm, 4.6×250 mm) was used for analysis at a flow rate of 1 ml min^−1^, to monitor the ligation reaction and analyze the purity of the peptide products. Ultimate XB-C4 or C18 column (Welch, 5 μm, 10×250 mm or 10 μm, 21.2×250 mm) were used to separate the ligation products and crude peptides, respectively, at a flow rate of 4–6 ml min^−1^. The purified products were characterized by ESI-MS on a Shimadzu LC/MS-2020 system. The CD spectra were obtained on an Applied Photophysics Chirascan-plus CD Spectrometer.

### Protein folding and purification

Lyophilized Dpo4-5m was dissolved in a denaturation buffer containing 6 m Gn·HCl, and dialyzed against a series of renaturation buffers which contained 4 m, 2 m, 1 m, 0.5 m, 0.25 m and 0 m Gn·HCl, respectively. Each step of the dialysis was carried out at 4 °C for 10 h with gentle stirring. The denaturation and renaturation buffers also contained 50 mm Tris-acetate (pH 7.5), 50 mm NaAc, 1 mm DTT, 0.5 mm EDTA and 16% glycerol. After renaturation, the enzyme was dialyzed against a buffer containing 10 mm potassium phosphate (pH 7.0), 50 mm NaCl, 10 mm MgAc_2_, 10% glycerol and 0.1% 2-Mercaptoethanol. The folded polymerase was incubated at 78 °C for 10 min to precipitate the thermolabile peptides, which were subsequently removed by ultracentrifugation at 19 000 r.p.m. for 40 min at 4 °C. The supernatant was incubated in Ni-NTA Superflow resin (Qiagen, Venlo, Netherlands) overnight at 4 °C, and purified according to previously described methods but without the use of Mono S column [18]. The concentration of the purified Dpo4-5m was measured spectrophotometrically at 280 nm using an extinction coefficient of 24 058 m^−1^cm^−1^ and M.W. of 40.8 kDa. Approximately 100 μg of the purified synthetic Dpo4-5m was analyzed by 12% SDS-PAGE along with the recombinant Dpo4-5m purified from the *E. coli* strain BL21(DE3).

### miPCR by synthetic d-Dpo4-5m

The l-DNA oligonucleotides were purchased from ChemGenes or synthesized by a Mermade 192E DNA/RNA synthesizer (BioAutomation, Irving, TX, USA). The PCR reactions were performed in 20 μl reaction systems containing 50 mm HEPES (pH 7.5), 5 mm MgCl_2_, 50 mm NaCl, 0.1 mm EDTA, 5 mm DTT, 10% glycerol, 3% DMSO, 0.1 mg ml^−1^ BSA, 100 μm (each) L-dNTPs, 0.5 μm (each) l-primers, 20 nm l-ssDNA template and ~500 nm d-Dpo4-5m polymerase. Because the mirror-image DNA polymerization was apparently less efficient than the corresponding natural system, likely due to the lower purity of l-dNTPs which was also observed in previous studies on the ASFV pol X system [1], an extension time of 1 h was used. The PCR program settings were 86 °C for 3 min (initial denaturation); 86 °C for 30 s (denaturation), 58 °C for 1 min (annealing) and 65 °C for 1 h (extension) for 40 cycles. The products were analyzed by 3% sieving agarose gel electrophoresis and stained by GoldView (Solarbio, China). Both the products of miPCR and PCR (5 μl) were digested by 0.5 U DNase I (NEB, Ipswich, MA, USA) at 37 °C for 10 min, or Exonuclease VIII, truncated (NEB) at 37 °C for 4 h, and analyzed by 3% sieving agarose gel electrophoresis.

## References

[bib1] Wang Z, Xu W, Liu L, Zhu TF. A synthetic molecular system capable of mirror-image genetic replication and transcription. Nat Chem 2016; 8: 698–704. 27325097 10.1038/nchem.2517

[bib2] Bohannon J. Mirror-image cells could transform science—or kill us all. Wired 2010. https://www.wired.com/2010/11/ff_mirrorlife/.

[bib3] Peplow M. Mirror-image enzyme copies looking-glass DNA. Nature 2016; 533: 303–304. 27193657 10.1038/nature.2016.19918

[bib4] Milton R, Milton S, Kent S. Total chemical synthesis of a D-enzyme: the enantiomers of HIV-1 protease show reciprocal chiral substrate specificity. Science 1992; 256: 1445–1448. 1604320 10.1126/science.1604320

[bib5] Weinstock MT, Jacobsen MT, Kay MS. Synthesis and folding of a mirror-image enzyme reveals ambidextrous chaperone activity. Proc Natl Acad Sci USA 2014; 111: 11679–11684. 25071217 10.1073/pnas.1410900111 PMC4136631

[bib6] Xu W, Jiang W, Wang J et al. Total chemical synthesis of a thermostable enzyme capable of polymerase chain reaction. Cell Discov 2017; 3: 17008. 28265464 10.1038/celldisc.2017.8 PMC5335361

[bib7] Pech A, Achenbach J, Jahnz M et al. A thermostable d-polymerase for mirror-image PCR. Nucleic Acids Res 2017; 45: 3997–4005. 28158820 10.1093/nar/gkx079 PMC5605242

[bib8] Dery L, Reddy PS, Dery S et al. Accessing human selenoproteins through chemical protein synthesis. Chem Sci 2017; 8: 1922–1926. 28451306 10.1039/c6sc04123j PMC5364654

[bib9] Dawson P, Muir T, Clark-Lewis I, Kent S. Synthesis of proteins by native chemical ligation. Science 1994; 266: 776–779. 7973629 10.1126/science.7973629

[bib10] Zheng J-S, Tang S, Qi Y-K, Wang Z-P, Liu L. Chemical synthesis of proteins using peptide hydrazides as thioester surrogates. Nat Protoc 2013; 8: 2483–2495. 24232250 10.1038/nprot.2013.152

[bib11] Yan LZ, Dawson PE. Synthesis of peptides and proteins without cysteine residues by native chemical ligation combined with desulfurization. J Am Chem Soc 2001; 123: 526–533. 11456564 10.1021/ja003265m

[bib12] Fang G-M, Li Y-M, Shen F et al. Protein chemical synthesis by ligation of peptide hydrazides. Angew Chem Int Ed Engl 2011; 50: 7645–7649. 21648030 10.1002/anie.201100996

[bib13] Huang Y-C, Fang G-M, Liu L. Chemical synthesis of proteins using hydrazide intermediates. Natl Sci Rev 2016; 3: 107–116.

[bib14] Williams KP, Liu X-H, Schumacher TNM et al. Bioactive and nuclease-resistant l-DNA ligand of vasopressin. Proc Natl Acad Sci 1997; 94: 11285–11290. 9326601 10.1073/pnas.94.21.11285 PMC23443

[bib15] Yatime L, Maasch C, Hoehlig K, Klussmann S, Andersen GR, Vater A. Structural basis for the targeting of complement anaphylatoxin C5a using a mixed L-RNA/L-DNA aptamer. Nat Commun 2015; 6: 6481. 25901944 10.1038/ncomms7481 PMC4423239

[bib16] Ling H, Boudsocq F, Woodgate R, Yang W. Crystal structure of a Y-family DNA polymerase in action: a mechanism for error-prone and lesion-bypass replication. Cell 2001; 107: 91–102. 11595188 10.1016/s0092-8674(01)00515-3

[bib17] Boudsocq F, Iwai S, Hanaoka F, Woodgate R. Sulfolobus solfataricus P2 DNA polymerase IV (Dpo4): an archaeal DinB-like DNA polymerase with lesion-bypass properties akin to eukaryotic polη. Nucleic Acids Res 2001; 29: 4607–4616. 11713310 10.1093/nar/29.22.4607 PMC92520

[bib18] Fiala KA, Suo Z. Pre-steady-state kinetic studies of the fidelity of Sulfolobus solfataricus P2 DNA polymerase IV. Biochemistry 2004; 43: 2106–2115. 14967050 10.1021/bi0357457

[bib19] Tucker WO, Shum KT, Tanner JA. G-quadruplex DNA Aptamers and their Ligands: Structure, Function and Application. Curr Pharm Des 2012; 18: 2014–2026. 22376117 10.2174/138161212799958477

[bib20] Jewett MC, Fritz BR, Timmerman LE, Church GM. *In vitro* integration of ribosomal RNA synthesis, ribosome assembly, and translation. Mol Syst Biol 2013; 9: 678. 23799452 10.1038/msb.2013.31 PMC3964315

[bib21] Stelzl U, Connell S, Nierhaus KH, Wittmann-Liebold B. Ribosomal proteins: role in ribosomal functions. eLS 2001. http://dx.doi.org/10.1038/npg.els.0000687.

[bib22] Pinheiro VB, Taylor AI, Cozens C et al. Synthetic genetic polymers capable of heredity and evolution. Science 2012; 336: 341–344. 22517858 10.1126/science.1217622 PMC3362463

[bib23] Larsen AC, Dunn MR, Hatch A, Sau SP, Youngbull C, Chaput JC. A general strategy for expanding polymerase function by droplet microfluidics. Nat Commun 2016; 7: 11235. 27044725 10.1038/ncomms11235 PMC4822039

[bib24] Chen T, Hongdilokkul N, Liu Z, Adhikary R, Tsuen SS, Romesberg FE. Evolution of thermophilic DNA polymerases for the recognition and amplification of C2'-modified DNA. Nat Chem 2016; 8: 556–562. 27219699 10.1038/nchem.2493 PMC4880425

[bib25] Jacobsen MT, Erickson PW, Kay MS. Aligator: a computational tool for optimizing total chemical synthesis of large proteins. Bioorg Med Chem 2017; 25: 4946–4952 28651912 10.1016/j.bmc.2017.05.061 PMC5860884

[bib26] Huang Y-C, Chen C-C, Li S-J, Gao S, Shi J, Li Y-M. Facile synthesis of C-terminal peptide hydrazide and thioester of NY-ESO-1 (A39-A68) from an Fmoc-hydrazine 2-chlorotrityl chloride resin. Tetrahedron 2014; 70: 2951–2955.

[bib27] White P, Keyte JW, Bailey K, Bloomberg G. Expediting the Fmoc solid phase synthesis of long peptides through the application of dimethyloxazolidine dipeptides. J Peptide Sci 2004; 10: 18–26. 14959888 10.1002/psc.484

[bib28] Coin I. The depsipeptide method for solid-phase synthesis of difficult peptides. J Peptide Sci 2010; 16: 223–230. 20401924 10.1002/psc.1224

