# Supplementary material for: Mirror-image polymerase chain reaction
Source: Cell Discov. 2017 Oct 17;3:17037–. doi: 10.1038/celldisc.2017.37 (PMC5643884; doi:10.1038/celldisc.2017.37)
Supplement: Supplementary Information [file celldisc201737-s1.pdf]

Supplementary Information

Mirror-image polymerase chain reaction

Wenjun Jiang[1,4], Baochang Zhang[2,4], Chuyao Fan[1,4], Min Wang[1,4], Jiaxing Wang[2], Qiang Deng[1], Xianyu Liu[1], Ji Chen[1], Jishen Zheng[3], Lei Liu[2*], Ting F. Zhu[1*]

[1]School of Life Sciences, Tsinghua-Peking Joint Center for Life Sciences, Center for Synthetic and Systems Biology, Ministry of Education Key Laboratory of Bioinformatics, Tsinghua University, Beijing 100084, China

[2]Tsinghua-Peking Joint Center for Life Sciences, Ministry of Education Key Laboratory of Bioorganic Phosphorus Chemistry and Chemical Biology, Department of Chemistry, Tsinghua University, Beijing 100084, China

[3]School of Life Sciences, University of Science and Technology of China, Hefei 230026, China

[4]These authors contributed equally to this work.

[*]To whom correspondence should be addressed. E-mail: tzhu@biomed.tsinghua.edu.cn (T.F.Z.); lliu@mail.tsinghua.edu.cn (L.L.)

Table of Contents Page

Supplementary Figures S1-S16 2
Supplementary Table S1 18

Supplementary Figures

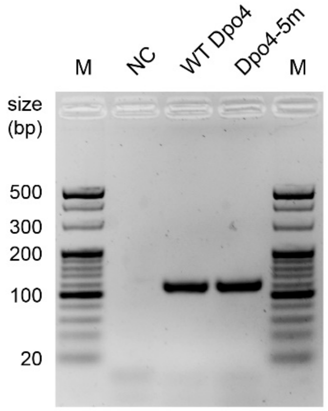

Supplementary Figure S1 | PCR by wild-type and mutant Dpo4. PCR amplification of a 120-bp sequence coding for the *E. coli* 5S rRNA sequence *rrfB* by recombinant WT Dpo4 and recombinant Dpo4-5m with five point mutations (C31S, S86C, N123A, S207A, and S313A), both purified from the *E. coli* strain BL21(DE3), performed in 50 mM HEPES (pH 7.5), 5 mM MgCl2, 50 mM NaCl, 0.1 mM EDTA, 5 mM DTT, 10% glycerol, 3% DMSO, 0.1 mg/ml BSA, 200 µM (each) dNTPs, 0.5 µM (each) primers, 2 nM template, and ~300 nM polymerase for 30 cycles. The products were analyzed by 3% sieving agarose gel electrophoresis and stained by GoldView. NC, negative control with template and primers but without enzyme. M, DNA marker.

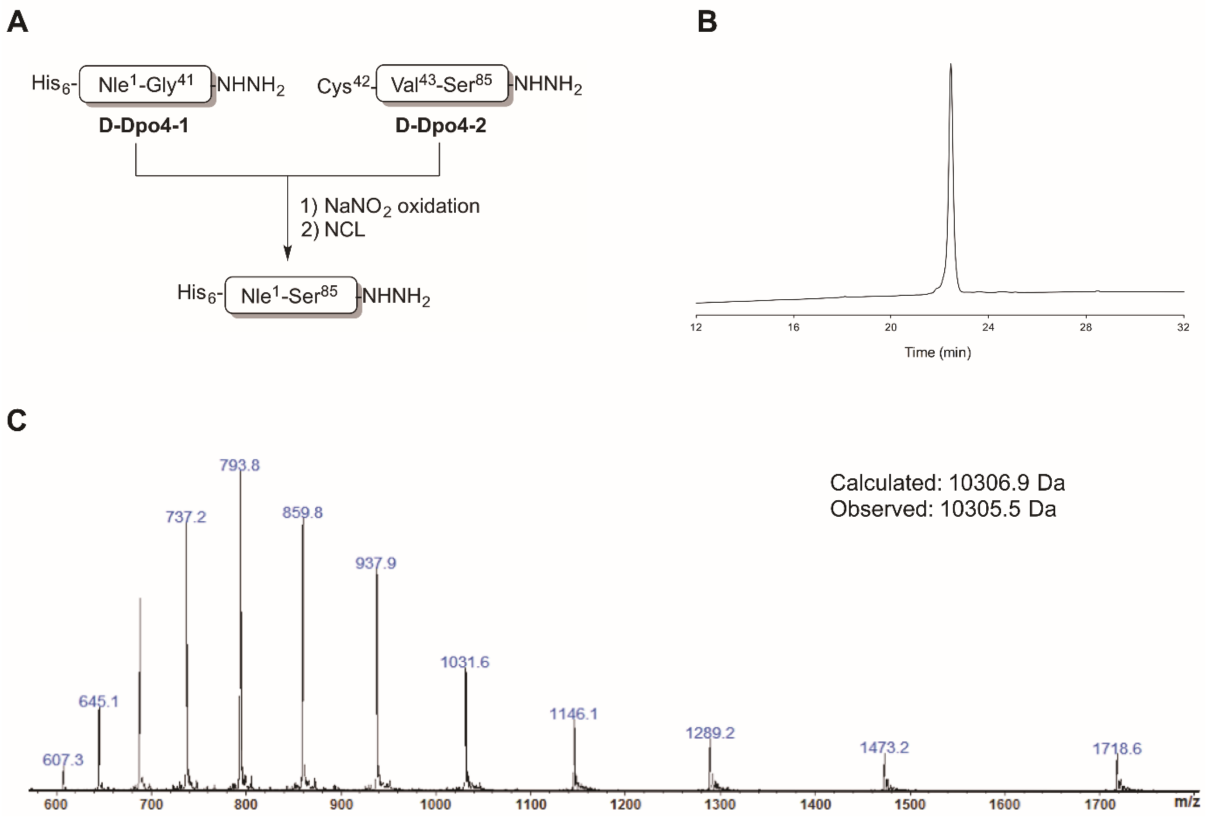

Supplementary Figure S2 | Ligation of D-Dpo4-1 and D-Dpo4-2. (**A**) D-Dpo4-1 (27 mg) was dissolved in 1 ml acidified ligation buffer (aqueous solution of 6 M Gn·HCl and 0.1 M NaH_2PO_4, pH 3.0). The mixture was cooled in ice-salt bath, and 100 µl 0.5 M $NaNO_2$ (in acidified ligation buffer) was added. The reaction was kept in ice-salt bath under stirring for 25 min, after which 1 ml 0.2 M MPAA (in 6 M Gn·HCl and 0.1 M Na_2HPO_4, pH 5.7) was added. After the addition of D-Dpo4-2 (24.5 mg), the pH of the reaction mixture was adjusted to 6.6 with NaOH solution at room temperature. After 15 h, the reaction mixture was reduced by TCEP and purified by HPLC (purification conditions: 20-80% CH_3CN (with 0.1% TFA) gradient in H_2O (with 0.1% TFA) over 30 min on a Welch C4 column). The ligation product was obtained with a yield of 39% (20 mg). (**B**) Analytical HPLC chromatogram of the ligation product of D-Dpo4-1 and D-Dpo4-2 (λ=214 nm). Column: Welch C4. Gradient: 20%-70% CH_3CN (with 0.1% TFA) in H_2O (with 0.1% TFA) over 30 min. (**C**) ESI-MS spectrum of the ligation product.

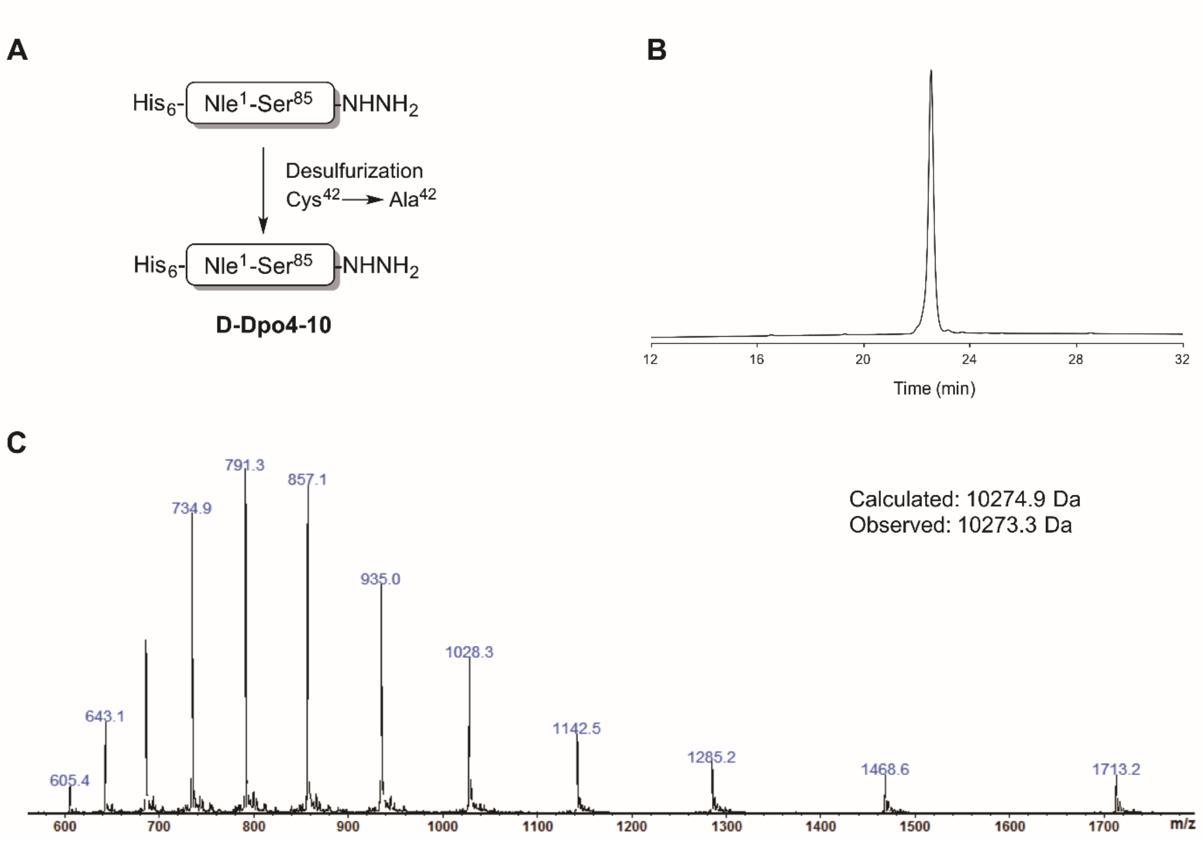

Supplementary Figure S3 | Preparation of D-Dpo4-10. (**A**) The ligation product of D-Dpo4-1 and D-Dpo4-2 (20 mg) was dissolved in 5 ml 200 mM TCEP solution (6 M Gn·HCl and 0.2 Na_2HPO_4, pH 6.8), with 0.1 mmol (32 mg) VA-044 and 0.2 mmol (62 mg) reduced L-glutathione added. The reaction was under stirring overnight at 37 °C. The desulfurization product Dpo4-10 was analyzed by HPLC and ESI-MS and purified by semi-preparative HPLC (purification conditions: 20-80% CH_3CN (with 0.1% TFA) gradient in H_2O (with 0.1% TFA) over 30 min on a Welch C4 column). After lyophilization, product D-Dpo4-10 was obtained with a yield of 79% (17 mg). (**B**) Analytical HPLC chromatogram of the desulfurization product D-Dpo4-10 (λ=214 nm). Column: Welch C4. Gradient: 20%-70% CH_3CN (with 0.1% TFA) in H_2O (with 0.1% TFA) over 30 min. (**C**) ESI-MS spectrum of D-Dpo4-10.

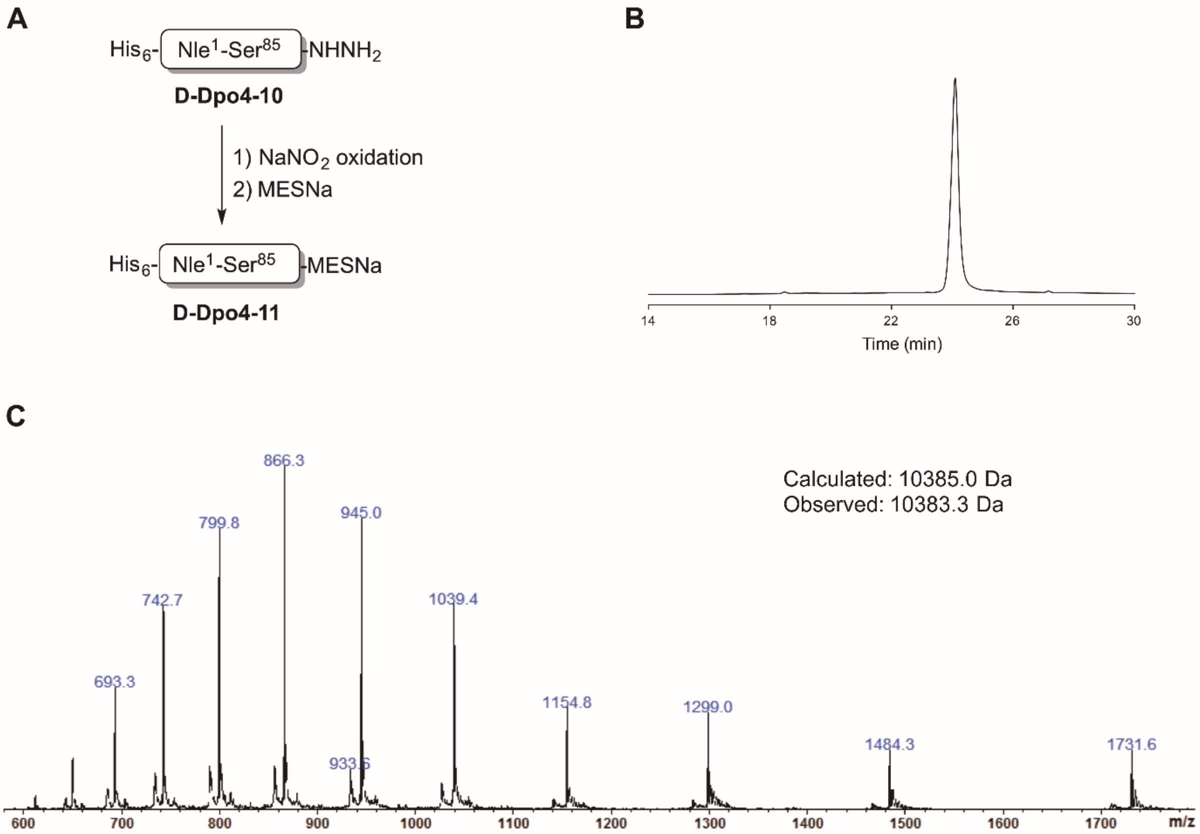

Supplementary Figure S4 | Preparation of D-Dpo4-11. (**A**) D-Dpo4-10 (15 mg) was dissolved in 0.7 ml acidified ligation buffer (aqueous solution of 6 M Gn·HCl and 0.1 M NaH_2PO_4, pH 3.0). The mixture was cooled in ice-salt bath, and 30 μl 0.5 M $NaNO_2$ (in acidified ligation buffer) was added. The reaction was kept in ice-salt bath under stirring for 25 min, after which 0.2 ml 0.4 M MESNa (in acidified ligation buffer) was added. The pH of the reaction mixture was adjusted to 5.0 with NaOH solution at room temperature. After 1 h, the products were purified by HPLC (purification conditions: 20-80% CH_3CN (with 0.1% TFA) gradient in H_2O (with 0.1% TFA) over 30 min on a Welch C4 column). D-Dpo4-11 was obtained with a yield of 82% (12.8 mg). (**B**) Analytical HPLC chromatogram of D-Dpo4-11 (λ=214 nm). Column: Welch C4. Gradient: 20%-70% CH_3CN (with 0.1% TFA) in H_2O (with 0.1% TFA) over 30 min. (**C**) ESI-MS spectrum of D-Dpo4-11.

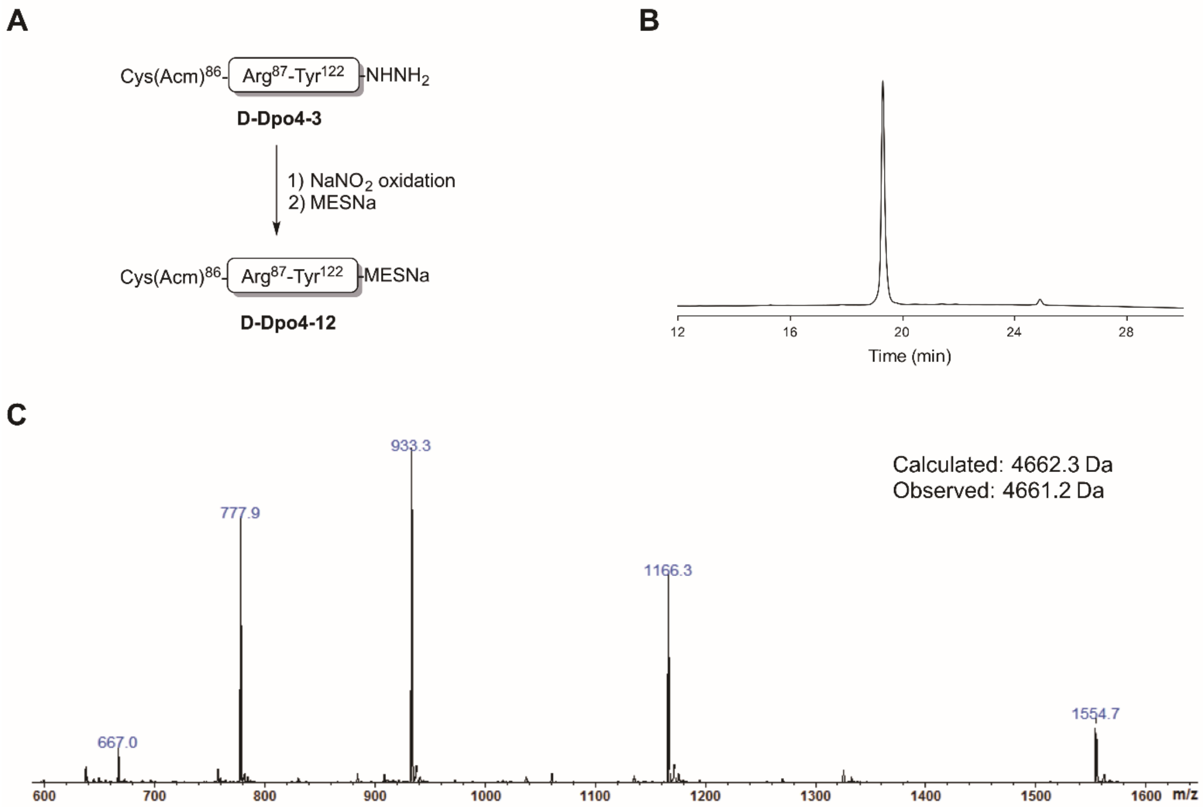

Supplementary Figure S5 | Preparation of D-Dpo4-12. (**A**) D-Dpo4-3 (23 mg) was dissolved in 2.5 ml acidified ligation buffer (aqueous solution of 6 M Gn·HCl and 0.1 M NaH_2PO_4, pH 3.0). The mixture was cooled in ice-salt bath, and 100 µl 0.5 M $NaNO_2$ (in acidified ligation buffer) was added. The reaction was kept in ice-salt bath under stirring for 25 min, after which 0.5 ml 0.4 M MESNa (in acidified ligation buffer) was added. The pH of the reaction mixture was adjusted to 4.8 with NaOH solution at room temperature. After 1 h, the products were purified by HPLC (purification conditions: 20-70% CH_3CN (with 0.1% TFA) gradient in H_2O (with 0.1% TFA) over 30 min on a Welch C4 column). D-Dpo4-12 was obtained with a yield of 56% (13 mg). (**B**) Analytical HPLC chromatogram of D-Dpo4-12 (λ=214 nm). Column: Welch C4. Gradient: 20%-70% CH_3CN (with 0.1% TFA) in H_2O (with 0.1% TFA) over 30 min. (**C**) ESI-MS spectrum of D-Dpo4-12.

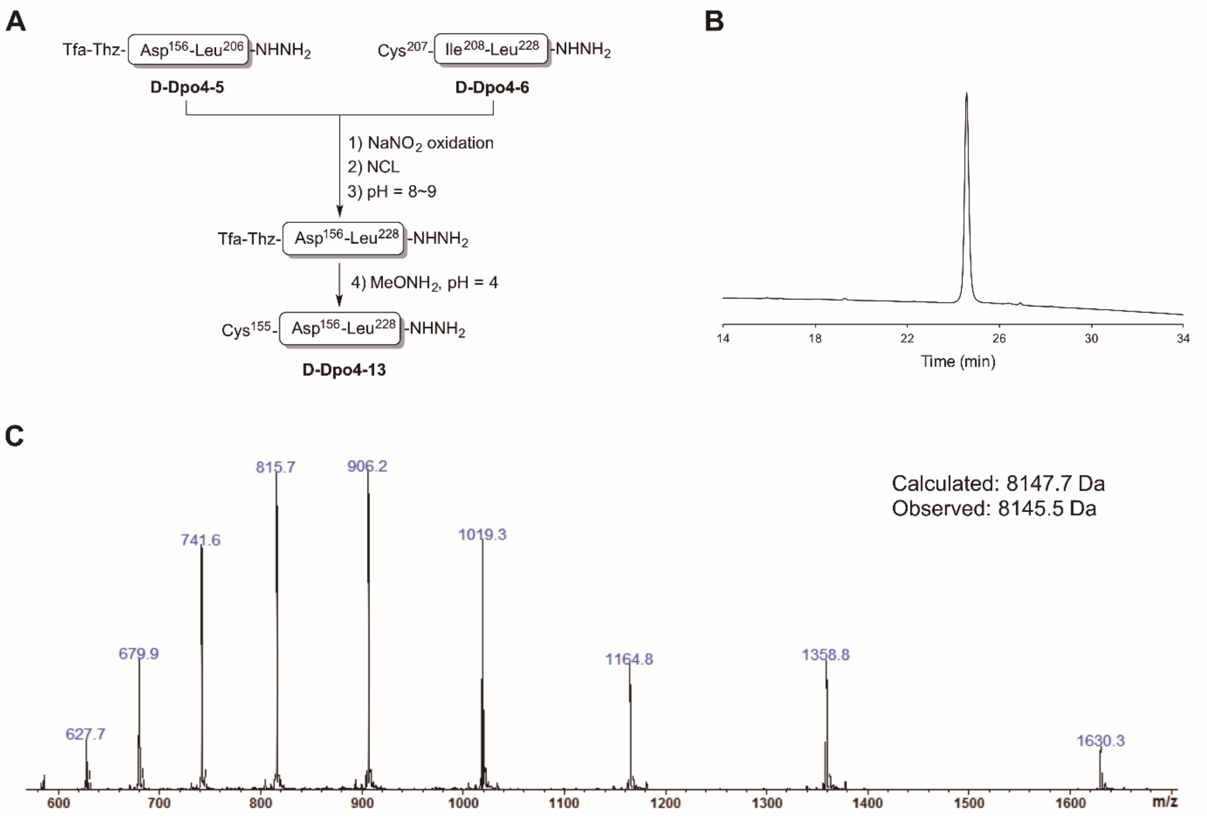

Supplementary Figure S6 | Preparation of D-Dpo4-13. (**A**) D-Dpo4-5 (23 mg) was dissolved in 0.8 ml acidified ligation buffer (aqueous solution of 6 M Gn·HCl and 0.1 M NaH_2PO_4, pH 3.0). The mixture was cooled in ice-salt bath, and 80 µl 0.5 M $NaNO_2$ (in acidified ligation buffer) was added. The reaction was kept in ice-salt bath under stirring for 25 min, after which 0.8 ml 0.2 M MPAA (in 6 M Gn·HCl and 0.1 M Na_2HPO_4, pH 5.8) was added. The pH of the reaction mixture was adjusted to 5.4 with NaOH solution at room temperature. After the addition of D-Dpo4-6 (12 mg), the pH of the reaction mixture was further adjusted to 6.6. After 14 h, the products were analyzed and the pH was adjusted to 8.8 to promote the complete removal of the Tfa group. After 1 h, the products were analyzed, and $MeONH_2$·HCl (24 mg) was added to carry out the conversion of Thz into Cys. TCEP·HCl was added until the pH of the reaction mixture reached 4.0. After 3 h, the products were analyzed and purified by HPLC (purification conditions: 20-80% CH_3CN (with 0.1% TFA) gradient in H_2O (with 0.1% TFA) over 30 min on a Welch C4 column). D-Dpo4-13 was obtained with a yield of 58% (19 mg). (**B**) Analytical HPLC chromatogram of D-Dpo4-13 (λ=214 nm). Column: Welch C4. Gradient: 20%-70% CH_3CN (with 0.1% TFA) in H_2O (with 0.1% TFA) over 30 min. (**C**) ESI-MS spectrum of D-Dpo4-13.

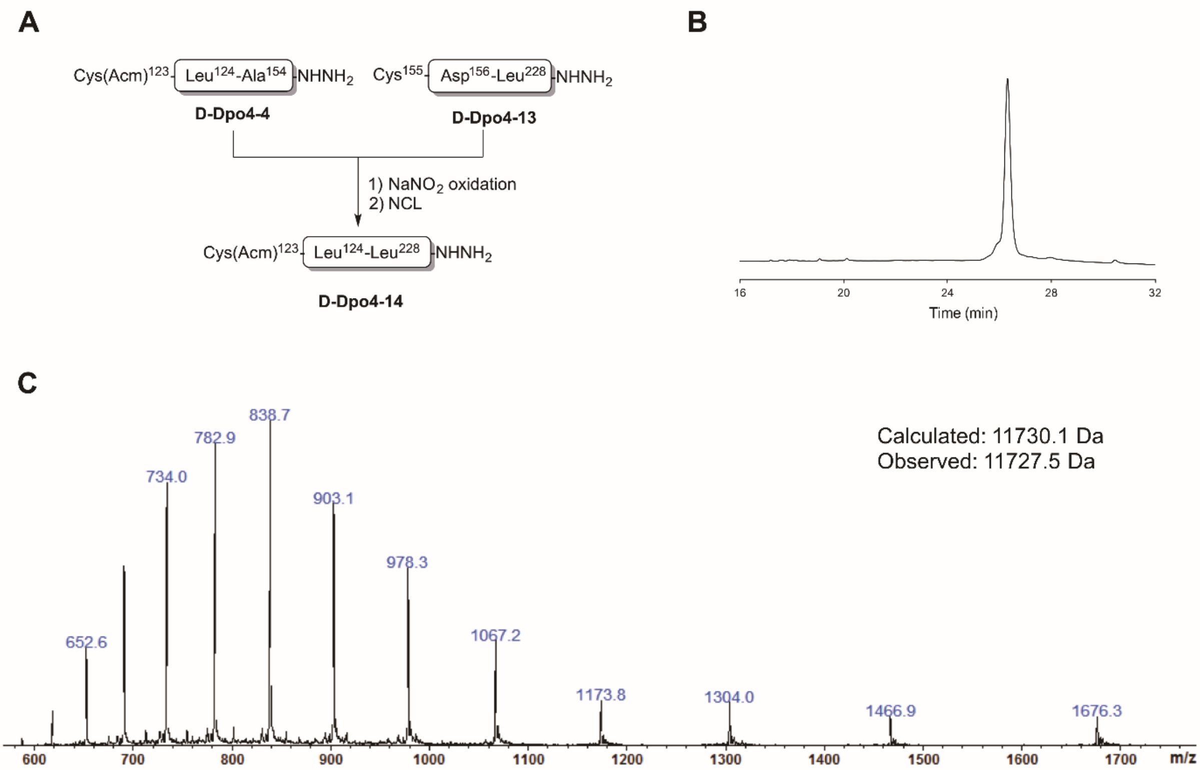

Supplementary Figure S7 | Preparation of D-Dpo4-14. (**A**) D-Dpo4-4 (20 mg) was dissolved in 2 ml acidified ligation buffer (aqueous solution of 6 M Gn·HCl and 0.1 M NaH_2PO_4, pH 3.0). The mixture was cooled in ice-salt bath, and 110 µl 0.5 M $NaNO_2$ (in acidified ligation buffer) was added. The reaction was kept in ice-salt bath under stirring for 25 min, after which 2 ml 0.22 M MPAA (in 6 M Gn·HCl and 0.1 M Na_2HPO_4, pH 5.7) was added. After the addition of D-Dpo4-13 (35 mg), the pH of the reaction mixture was adjusted to 6.6 with NaOH solution at room temperature. After 15 h, the reaction mixture was reduced by TCEP and purified by HPLC (purification conditions: 20-70% CH_3CN (with 0.1% TFA) gradient in H_2O (with 0.1% TFA) over 30 min on a Welch C4 column). D-Dpo4-14 was obtained with a yield of 43% (22 mg). (**B**) Analytical HPLC chromatogram of D-Dpo4-14 (λ=214 nm). Column: Welch C4. Gradient: 20%-70% CH_3CN (with 0.1% TFA) in H_2O (with 0.1% TFA) over 30 min. (**C**) ESI-MS spectrum of D-Dpo4-14.

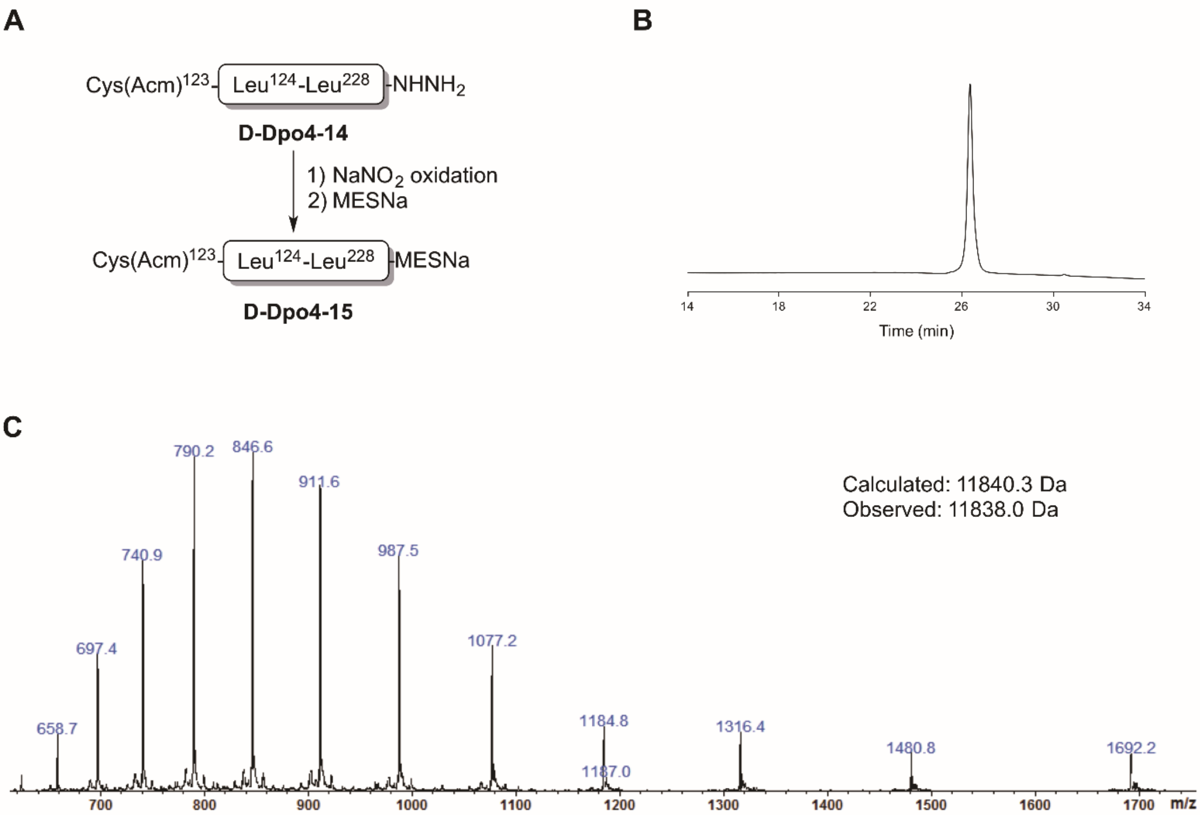

Supplementary Figure S8 | Preparation of D-Dpo4-15. (**A**) D-Dpo4-14 (58 mg) was dissolved in 4.9 ml acidified ligation buffer (aqueous solution of 6 M Gn·HCl and 0.1 M NaH_2PO_4, pH 3.0). The mixture was cooled in ice-salt bath, and 98 µl 0.5 M $NaNO_2$ (in acidified ligation buffer) was added. The reaction was kept in ice-salt bath under stirring for 25 min, after which 1.2 ml 0.8 M MESNa (in acidified ligation buffer) was added. The pH of the reaction mixture was adjusted to 5.5 with NaOH solution at room temperature. After 1 h, the products were analyzed by HPLC and reduced by TCEP before purification (purification conditions: 20-80% CH_3CN (with 0.1% TFA) gradient in H_2O (with 0.1% TFA) over 30 min on a Welch C4 column). D-Dpo4-15 was obtained with a yield of 79% (46 mg). (**B**) Analytical HPLC chromatogram of D-Dpo4-15 (λ=214 nm). Column: Welch C4. Gradient: 20%-70% CH_3CN (with 0.1% TFA) in H_2O (with 0.1% TFA) over 30 min. (**C**) ESI-MS spectrum of D-Dpo4-15.

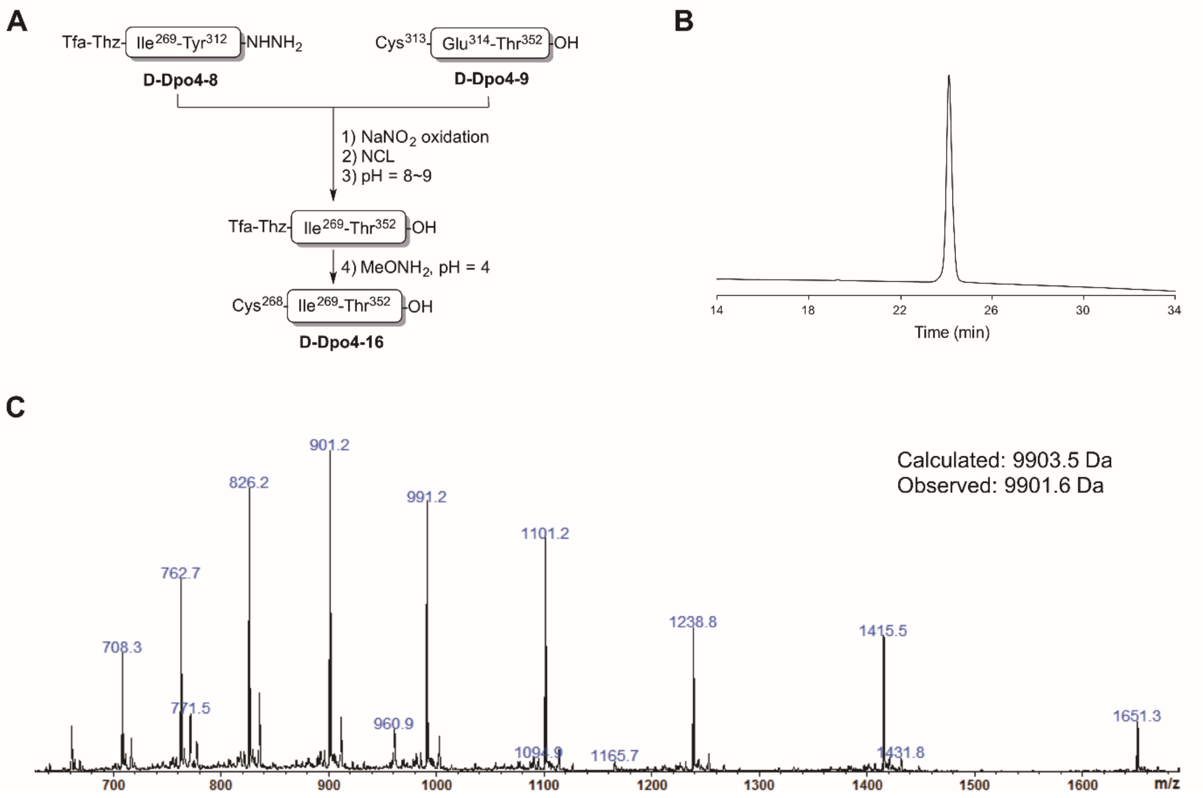

Supplementary Figure S9 | Preparation of D-Dpo4-16. (**A**) The ligation of D-Dpo4-8 (53 mg) with D-Dpo4-9 (48 mg) was carried out following a procedure similar to the ligation of D-Dpo4-5 with D-Dpo4-6 (see Supplementary Figure S6). Purification conditions: 15%-95% CH_3CN (with 0.1% TFA) gradient in H_2O (with 0.1% TFA) over 30 min on a Welch C4 column). D-Dpo4-16 was obtained with a yield of 46% (46 mg). (**B**) Analytical HPLC chromatogram of D-Dpo4-16 (λ=214 nm). Column: Welch C4. Gradient: 20%-70% CH_3CN (with 0.1% TFA) in H_2O (with 0.1% TFA) over 30 min. (**C**) ESI-MS spectrum of D-Dpo4-16.

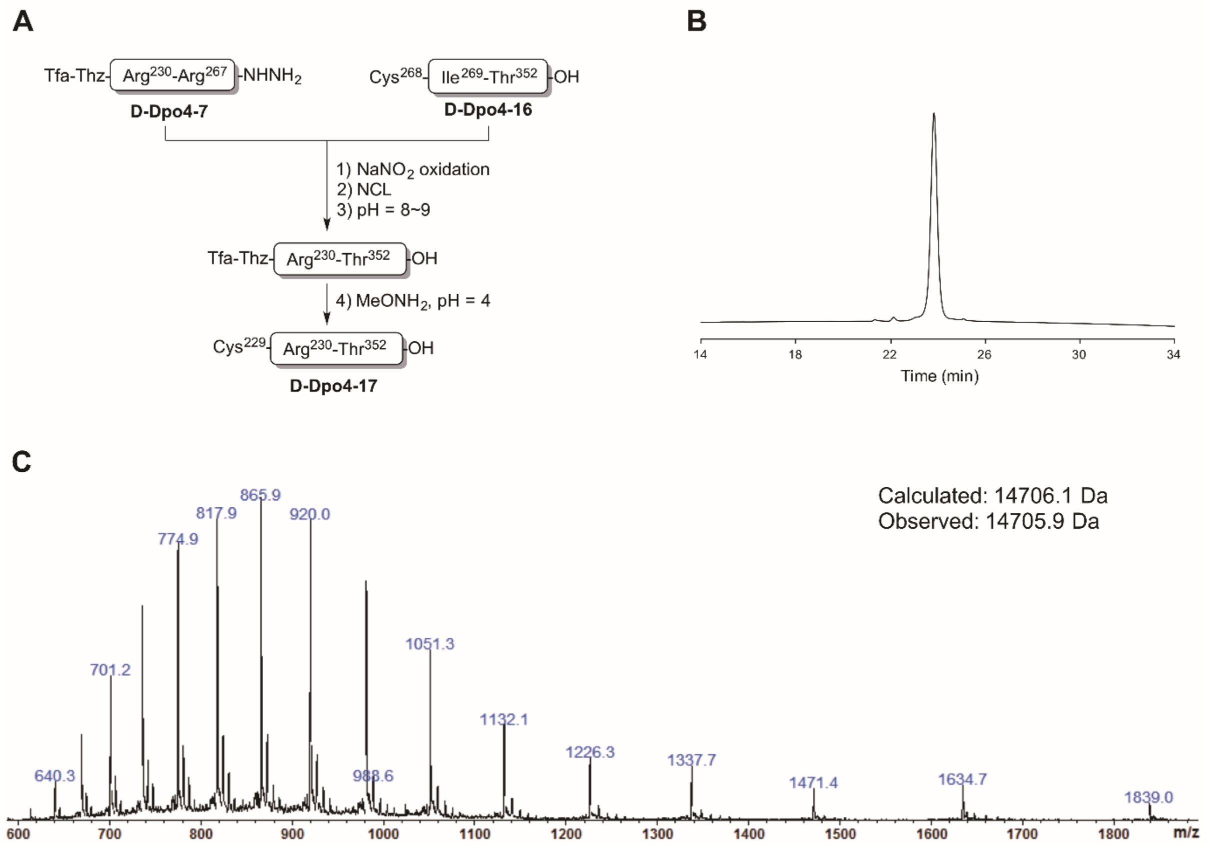

Supplementary Figure S10 | Preparation of D-Dpo4-17. (**A**) The ligation of D-Dpo4-7 (30 mg) with D-Dpo4-16 (48 mg) was carried out following a procedure similar to the ligation of D-Dpo4-5 with D-Dpo4-6 (see Supplementary Figure S6). Purification conditions: 20%-70% CH_3CN (with 0.1% TFA) gradient in H_2O (with 0.1% TFA) over 30 min on a Welch C4 column). D-Dpo4-17 was obtained with a yield of 50% (36 mg). (**B**) Analytical HPLC chromatogram of D-Dpo4-17 (λ=214 nm). Column: Welch C4. Gradient: 20%-70% CH_3CN (with 0.1% TFA) in H_2O (with 0.1% TFA) over 30 min. (**C**) ESI-MS spectrum of D-Dpo4-17.

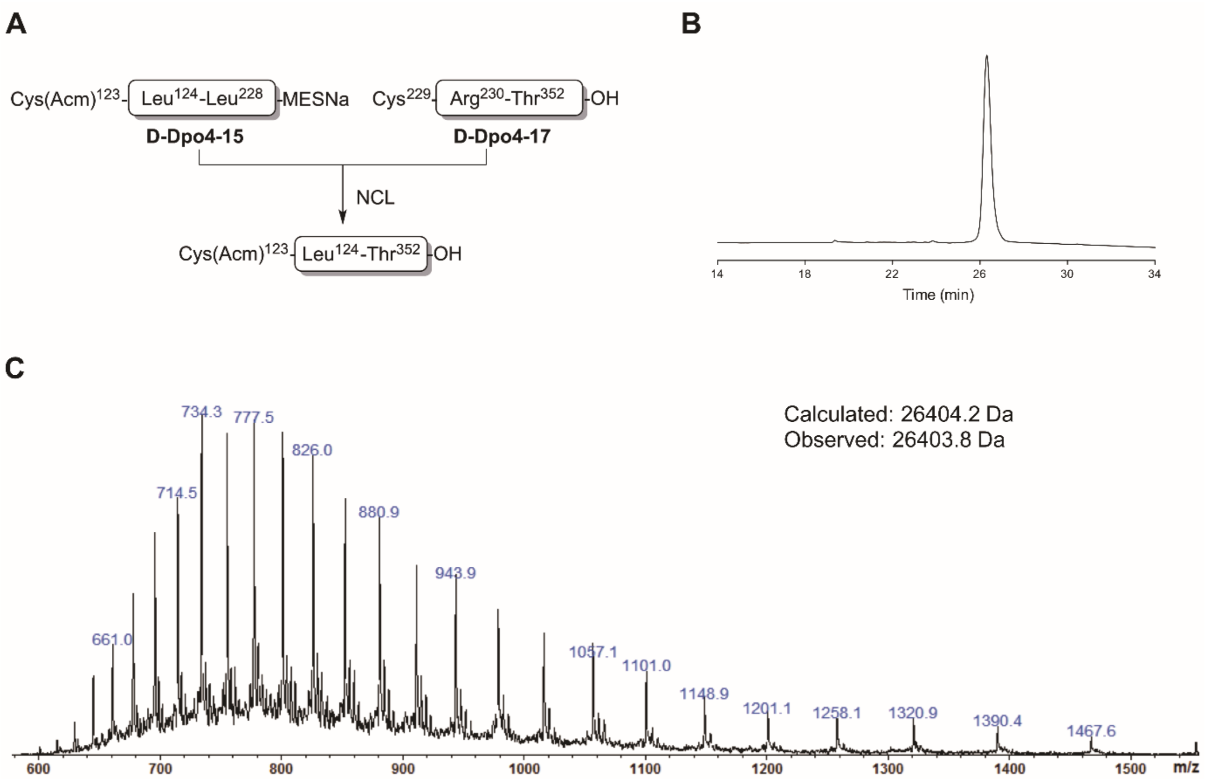

Supplementary Figure S11 | Ligation of D-Dpo4-15 and D-Dpo4-17. (**A**) D-Dpo4-15 (33 mg) and D-Dpo4-17 (36 mg) were dissolved in a 1.2 ml aqueous solution of 6 M Gn·HCl, 0.1 M Na_2HPO_4, 40 mM TCEP and 125 mM MPAA, pH 6.8. The pH of the reaction mixture was adjusted to 6.8. After 18 h, the products were analyzed by HPLC, after which the products were diluted and purified (purification conditions: 20-80% CH_3CN (with 0.1% TFA) gradient in H_2O (with 0.1% TFA) over 30 min on a Welch C4 column). Dpo4-18 was obtained with a yield of 82% (53 mg). (**B**) Analytical HPLC chromatogram of the ligation product of D-Dpo4-15 and D-Dpo4-17 (λ=214 nm). Column: Welch C4. Gradient: 20%-70% CH_3CN (with 0.1% TFA) in H_2O (with 0.1% TFA) over 30 min. (**C**) ESI-MS spectrum of the ligation product.

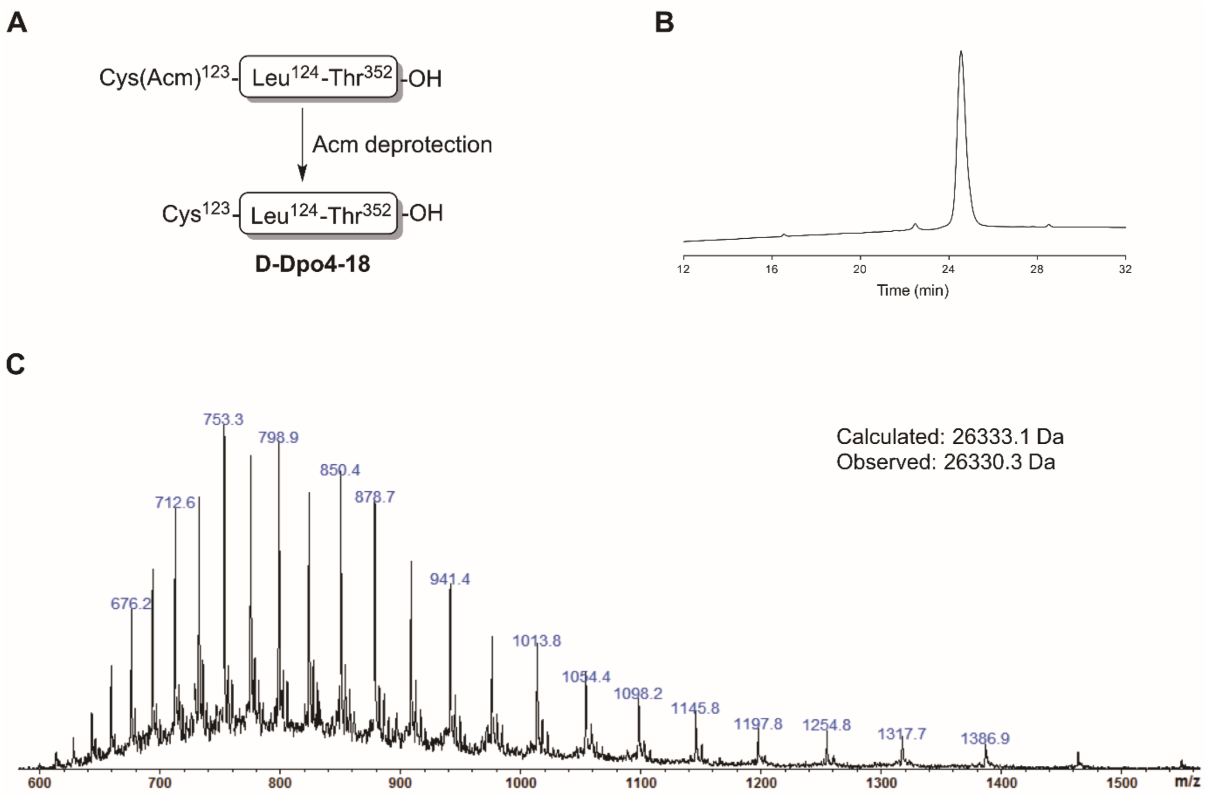

Supplementary Figure S12 | Preparation of D-Dpo4-18. (**A**) The ligation product of D-Dpo4-15 and D-Dpo4-17 (49 mg) was dissolved in a 1.8 ml aqueous solution of 6 M Gn·HCl, 0.1 M Na_2HPO_4 and 40 mM TCEP, pH 7.1. $PdCl_2$ (9.5 mg) was dissolved in a 0.36 ml aqueous solution of 6 M Gn·HCl and 0.1 M Na_2HPO_4 and added to the peptide solution. After 16 h, 4 ml 0.75 M DTT (in an aqueous solution of 6 M Gn·HCl and 0.1 M Na_2HPO_4) was added. The reaction mixture was under stirring for 30 min and purified by HPLC (purification conditions: 20-80% CH_3CN (with 0.1% TFA) gradient in H_2O (with 0.1% TFA) over 30 min on a Welch C4 column). D-Dpo4-18 was obtained with a yield of 86% (42 mg). (**B**) Analytical HPLC chromatogram of D-Dpo4-18 (λ=214 nm). Column: Welch C4. Gradient: 20%-70% CH_3CN (with 0.1% TFA) in H_2O (with 0.1% TFA) over 30 min. (**C**) ESI-MS spectrum of D-Dpo4-18.

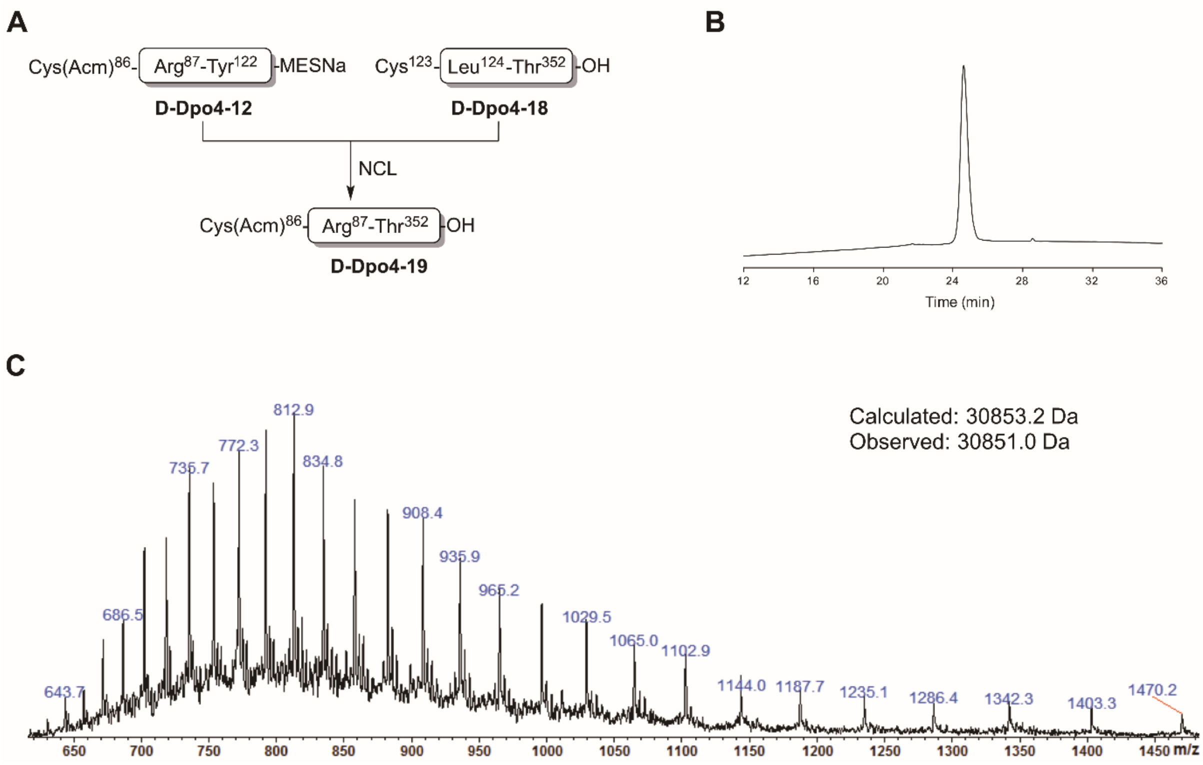

Supplementary Figure S13 | Preparation of D-Dpo4-19. (**A**) D-Dpo4-12 (17 mg) and D-Dpo4-18 (42 mg) were dissolved in a 1.6 ml aqueous solution of 6 M Gn·HCl, 0.1 M Na_2HPO_4, 40 mM TCEP and 100 mM MPAA, pH 6.8. The pH of the reaction mixture was adjusted to 6.6. After 15 h, the products were analyzed by HPLC, diluted, and purified by HPLC (purification conditions: 20%-80% CH_3CN (with 0.1% TFA) gradient in H_2O (with 0.1% TFA) over 30 min on a Welch C4 column). D-Dpo4-19 was obtained with a yield of 86% (43 mg). (**B**) Analytical HPLC chromatogram of D-Dpo4-19 (λ=214 nm). Column: Welch C4. Gradient: 20%-70% CH_3CN (with 0.1% TFA) in H_2O (with 0.1% TFA) over 30 min. (**C**) ESI-MS spectrum of D-Dpo4-19.

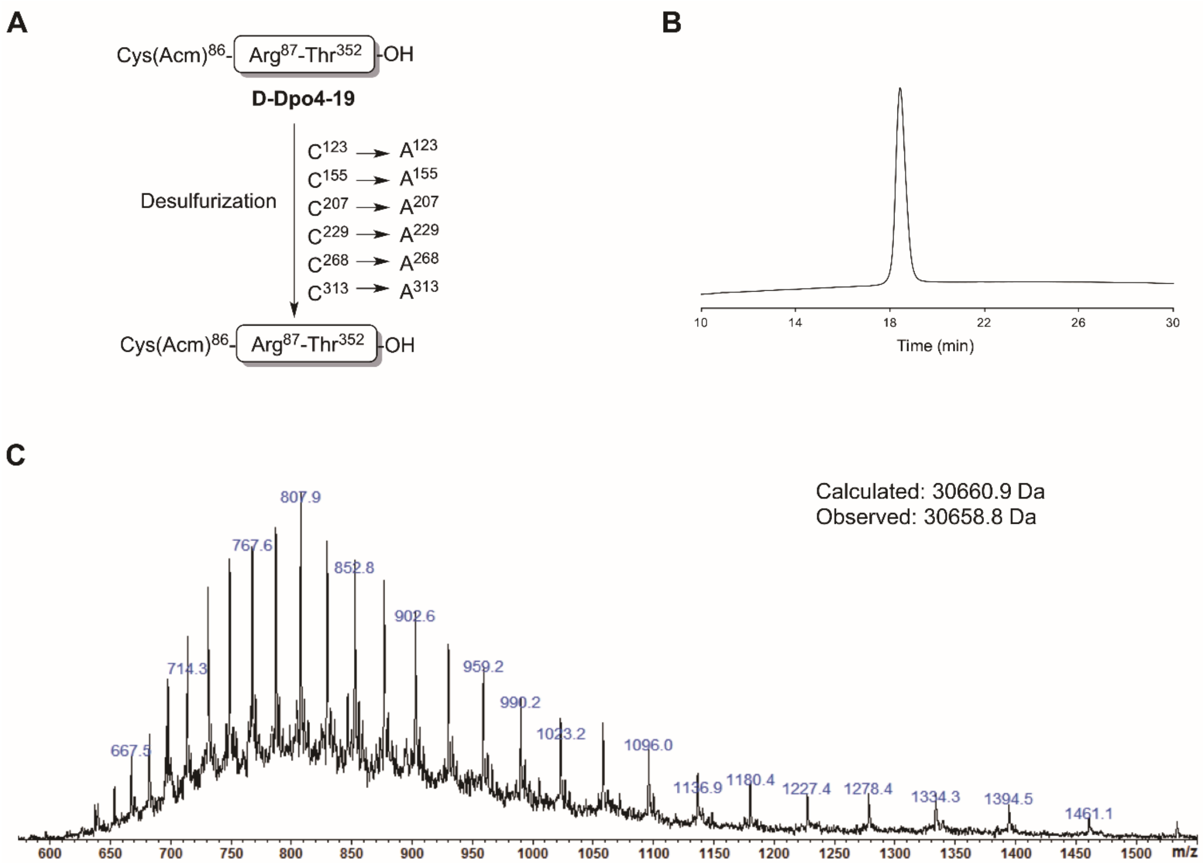

Supplementary Figure S14 | Desulfurization of D-Dpo4-19. (**A**) D-Dpo4-19 (42.5 mg) was dissolved in 2.3 ml aqueous solution of 6 M Gn·HCl and 0.1 M Na_2HPO_4, Subsequently, 9 ml 380 mM TCEP (in 6 M Gn·HCl and 0.1 M Na_2HPO_4, pH 6.8), 0.46 mmol (140 mg) reduced L-glutathione and 0.23 mmol (73 mg) VA-044 were added. The reaction was under stirring overnight at 37 °C. The desulfurization product of D-Dpo4-19 was analyzed by HPLC and ESI-MS, and purified by semi-preparative HPLC (purification conditions: 20%-80% CH_3CN (with 0.1% TFA) gradient in H_2O (with 0.1% TFA) over 30 min on a Welch C4 column). After lyophilization, the desulfurization product was obtained with a yield of 76% (32.5 mg). (**B**) Analytical HPLC chromatogram of the desulfurization product of D-Dpo4-19 (λ=214 nm). Column: Welch C4. Gradient: 20%-70% CH_3CN (with 0.1% TFA) in H_2O (with 0.1% TFA) over 30 min. (**C**) ESI-MS spectrum of the desulfurization product.

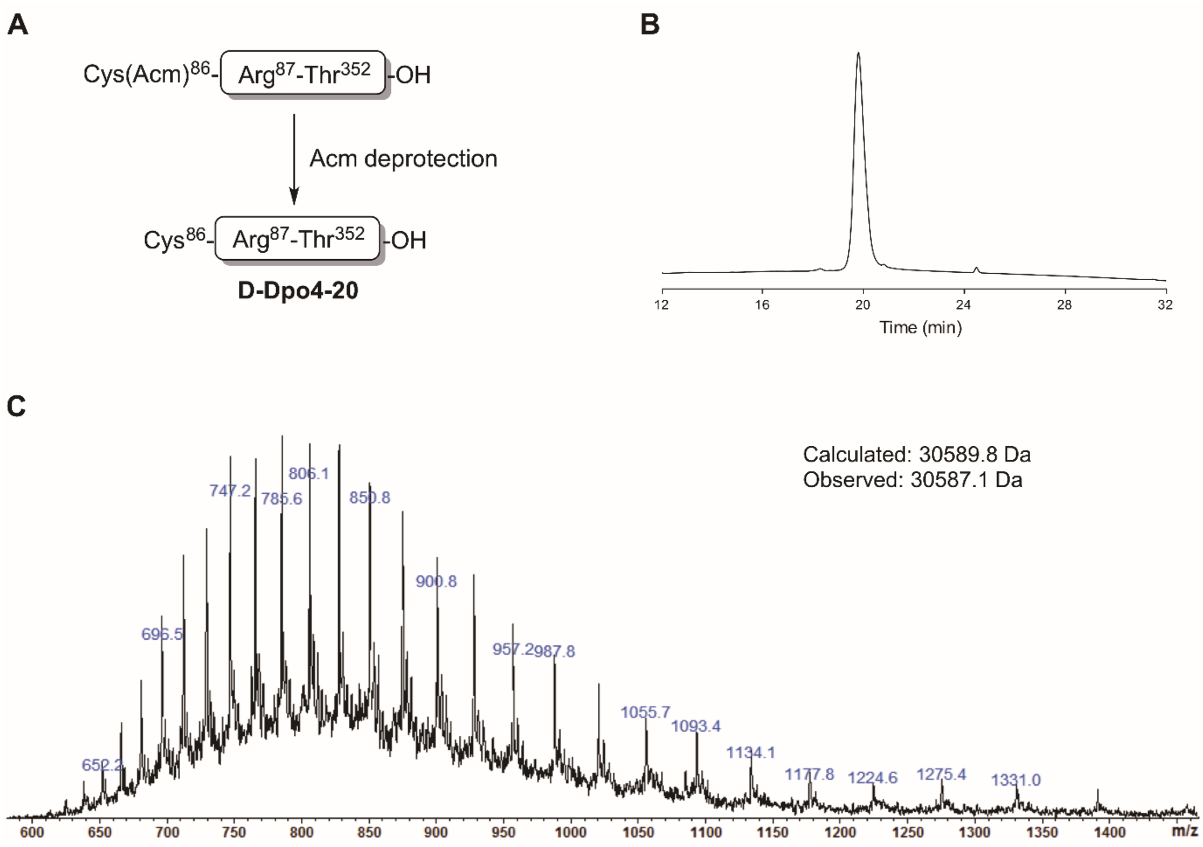

Supplementary Figure S15 | Preparation of D-Dpo4-20. (**A**) The desulfurization product of D-Dpo4-19 (32.5 mg) was dissolved in a 2.2 ml 50% acetic acid aqueous solution. Subsequently, 16 mg silver acetate was added to the solution. The reaction was under stirring overnight. After 12 h, 0.9 ml 7 M 2-Mercaptoethanol (in aqueous solution of 6 M Gn·HCl and 0.1 M Na_2HPO_4) was added. The system was diluted with 4 ml ligation buffer (6 M Gn·HCl, 0.1 M Na_2HPO_4, pH=7.4). After centrifugation, the supernatant was purified by semi-preparative HPLC. The precipitant was washed thoroughly and purified (purification conditions: 20%-80% CH_3CN (with 0.1% TFA) gradient in H_2O (with 0.1% TFA) over 30 min on a Welch C4 column). After lyophilization, the Acm-removed product D-Dpo4-20 was obtained with a yield of 81% (26 mg). (**B**) Analytical HPLC chromatogram of D-Dpo4-20 (λ=214 nm). Column: Welch C4. Gradient: 30%-80% CH_3CN (with 0.1% TFA) in H_2O (with 0.1% TFA) over 30 min. (**C**) ESI-MS spectrum of D-Dpo4-20.

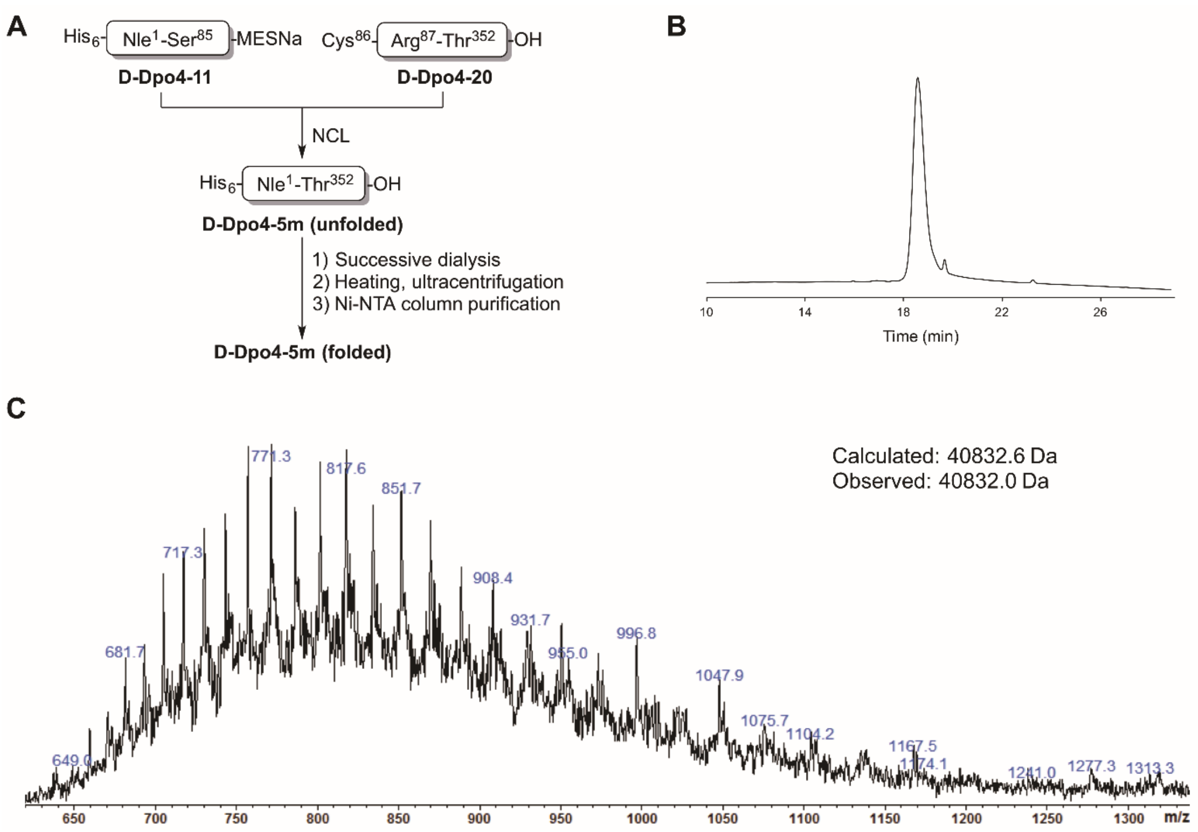

Supplementary Figure S16 | Preparation of D-Dpo4-5m. (**A**) D-Dpo4-11 (6.3 mg) and D-Dpo4-20 (9.2 mg) were dissolved in 0.3 ml aqueous solution of 6 M Gn·HCl, 0.1 M Na_2HPO_4, 80 mM MPAA and 40 mM TCEP, pH 6.6. Subsequently, the pH of the reaction mixture was adjusted to 6.6. After 16 h, the system was analyzed and diluted with 0.3 ml 100 mM TCEP (in aqueous solution of 6 M Gn·HCl and 0.1 M Na_2HPO_4, pH 6.4). The product was purified by HPLC (purification conditions: 20%-70% CH_3CN (with 0.1% TFA) gradient in H_2O (with 0.1% TFA) over 30 min on a Welch C4 column). Unfolded D-Dpo4-5m was obtained with a yield of 77% (9.5 mg). The product then underwent successive dialysis, precipitation of thermolabile peptides, purification by Ni-NTA column, and concentration by centrifugal filter. The folded synthetic D-Dpo4-5m was obtained with a yield of ~15%. (**B**) Analytical HPLC chromatogram of unfolded synthetic D-Dpo4-5m (λ=214 nm). Column: Welch C4. Gradient: 30%-80% CH_3CN (with 0.1% TFA) in H_2O (with 0.1% TFA) over 30 min. (**C**) ESI-MS spectrum of unfolded synthetic D-Dpo4-5m.

Supplementary Table S1 | DNA oligo sequences

D-/L-DNA oligo	Sequence
D-/L-5S rRNA(*rrfB*)-T	5'-ATGCCTGGCAGTTCCCTACTCTCGCATGGGGAGACCCCACACTACCATCGGCGCTACGGCGTTTCACTTCTGAGTTCGGCATGGGGTCAGGTGGGACCACCGCGCTACTGCCGCCAGGCA -3'
D-/L-5S rRNA(*rrfB*)-F	5'-TGCCTGGCGGCAGTAGCGC-3'
D-/L-5S rRNA(*rrfB*)-R	5'-ATGCCTGGCAGTTCCCTACTCTCGC-3'
